# Deep Learning and Entropy-Based Texture Features for Color Image Classification

**DOI:** 10.3390/e24111577

**Published:** 2022-10-31

**Authors:** Emma Lhermitte, Mirvana Hilal, Ryan Furlong, Vincent O’Brien, Anne Humeau-Heurtier

**Affiliations:** 1 Univ Angers, LARIS, SFR MATHSTIC, F-49000 Angers, France; 2 Institute of Technology Carlow, R93 V960 Carlow, Ireland

**Keywords:** biomedical data, classification, deep learning, entropy, RGB images, texture

## Abstract

In the domain of computer vision, entropy—defined as a measure of irregularity—has been proposed as an effective method for analyzing the texture of images. Several studies have shown that, with specific parameter tuning, entropy-based approaches achieve high accuracy in terms of classification results for texture images, when associated with machine learning classifiers. However, few entropy measures have been extended to studying color images. Moreover, the literature is missing comparative analyses of entropy-based and modern deep learning-based classification methods for RGB color images. In order to address this matter, we first propose a new entropy-based measure for RGB images based on a multivariate approach. This multivariate approach is a bi-dimensional extension of the methods that have been successfully applied to multivariate signals (unidimensional data). Then, we compare the classification results of this new approach with those obtained from several deep learning methods. The entropy-based method for RGB image classification that we propose leads to promising results. In future studies, the measure could be extended to study other color spaces as well.

## 1. Introduction

Texture analysis of an image is a key concept in computer vision. The texture analysis can be applied in multiple fields, such as object recognition, pattern recognition, and biomedical images classification, among others. Some of the most recently developed methods that describe the texture of an image are based on information theory concepts and, more precisely, on entropy measures [1]. Entropy quantifies the irregularity: it increases with the degree of disorder and is maximal for completely random systems.

In the recent decade, we have seen the emergence of multiple variations of entropy-based measures. One-dimensional (1D) entropy measures (e.g., approximate entropy 1D [2], sample entropy 1D [3], and fuzzy entropy 1D [4]) have shown that, by quantifying the irregularity of biological temporal series, it is possible to differentiate healthy from diseased systems and to classify them with a high degree of accuracy. With the work of Ahmed et al. [5], entropy-based measures for multivariate signals, which we often encounter in biological systems, have been developed [6]. For instance, it has been possible to identify women at risk of preterm delivery by classifying uterine electromyogram recordings from their multivariate entropy values [7]. Moreover, most of the above-mentioned unidimensional entropy methods have been extended to their multiscale version [6,7,8]. With the development of two-dimensional (2D) versions of entropy algorithms (e.g., bi-dimensional sample entropy [9], bi-dimensional fuzzy entropy [10], bi-dimensional permutation entropy [11], and bi-dimensional dispersion entropy [12]), designed to extract texture information from 2D patterns in grayscale images, it has been possible to successfully classify biomedical images from healthy and pathological subjects, such as dermoscopic images or cells images [10].

Before the breakthrough of data-driven methods to classify images in the last decade, extracting image features by hand was the most frequently used method. For images, the feature, or vector of features, is a representation of the image that can be used for classification purposes using machine learning classifiers [13]. One of the limitations of machine learning-based methods is that machine learning classifiers cannot process raw data (such as images). The drawback of traditional machine learning approaches, whereby the feature vectors have to be created by experts, is addressed by deep learning. In the latter, the feature vectors are not designed by humans, but learned automatically from the data [14]. The applications of such methods are multiple, e.g., object detection, face identification, or texture analysis. In 2012, Krizhevsky et al. have been able to develop a very large network and to train it with a lot of data from the ImageNet dataset [15]. This has been performed without overfitting, thanks to innovative techniques. The model achieved the best performances with a reduced training time at that time. This paper inspired many models widely used today, such as VGG networks [16], residual networks [17], and dense networks [18].

Despite all the texture extraction methods that have been developed, far less have been proposed for color RGB images [19,20,21]. Moreover, very few of them are based on entropy measures [22]. When deep learning-based approaches are used on color images, good accuracy is usually obtained in classification results. However, the learning-based approaches have the drawback of relying on a training step and of being black boxes for the end user. Analyzing those two different approaches (entropy-based measures and deep learning methods) would allow us to compare their performances and to determine the advantages and drawbacks of each of them for color images.

In this paper, we propose to study texture for color images through two approaches: the first one is a hand-crafted feature extraction method; the second one uses the deep learning approach. For the hand-crafted method, we focus on entropy-based measures because of their novelty [19] and the promising results they have shown for grayscale images. Moreover, extensions of these measures to color images have not been studied thoroughly yet. We first propose two new approaches of entropy-based measures to analyze the texture of RGB images: the multivariate multiscale sample entropy and the multivariate multiscale fuzzy entropy ($MMSampEn_{RGB}$ and $MMFuzEn_{RGB}$, respectively). We detail their validation tests, application to biomedical texture images, and comparison to other texture classification methods. Then, we propose to compare entropy-based methods with deep learning approaches for texture image classification. Our experimentation will explore the classification results of different neural networks architectures. This work will complement other comparative studies present in the literature ([21,23,24]) with a more precise approach since it focuses on entropy measures.

The paper is organized as follows: first, we present the datasets and methods used to evaluate our algorithms. Then, the different existing and new entropy-based methods are presented, followed by the description of the implemented deep learning architectures. Finally, we present the comparison of the results obtained and discuss them.

## 2. Materials and Methods

### 2.1. Datasets

The classification has been performed on a biomedical texture database (Epistroma), generic texture databases (KTH-TIPS2-a and Alot), and synthetic images (MIX process). All the experimental results were obtained using Matlab 2020a software and Python3 installed in Intel Xeon(R) W-2223 processor, NV137 graphics card laptop.

The Epistroma dataset [25] is composed of images of epithelium (825 samples) and stroma (551 samples) tissues samples from a series of 643 consecutive patients who underwent surgery for histologically verified colorectal cancer at the Helsinki University Central Hospital between 1989 and 1998. Linder et al. [26] showed that the texture analysis is a relevant method to discriminate the two histological tissue types.

The KTH-TIPS2-a dataset [27] is used to evaluate the ability of our algorithm in identifying categories of textures such as “wood” or “wool” with different illuminations and poses [28]. The database contains 4 physical samples of 11 different materials. For more details on the databases, see Table 1.

The Alot dataset [29] is composed of 25,000 images of different materials. The singularity of this dataset is that the captured images are material-specific representations of 250 materials. The distinctive properties of the materials are highlighted with the variation of imaging settings, such as local intensity variation or color properties [30]. This adjustment to each image makes it easier to distinguish one type of material from the others.

### 2.2. Synthetic Images

#### 2.2.1. MIX Process for Multivariate One-Dimensional Signals

**Figure 1:**
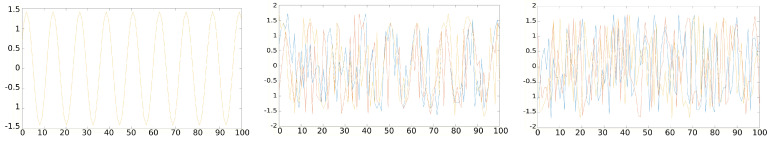
First 100 samples of a multivariate signal generated with a MIX process. *p* varies from 0 to 1 with a step of 0.5 (from left to right).

In order to evaluate entropy extraction algorithms of signals composed of *q* variates, we introduce a MIX process based on the one-dimensional version. The one-dimensional version, MIX(p), is defined as a family of one-dimensional stochastic processes that generates signals by asserting, for each point, whether the latter belongs to a deterministic sine function or to a random uniform variable, according to the choice of the parametric probability *p*, 0<p<1 [2]. The larger the *p* value is, the more frequent the random dynamics are. We define the MIX process for multivariate signals with *q* variates as follows: let $X_{i}=\sin\frac{2\pi i}{12}$ be a sinusoidal signal, and $Y_{i}$ uniform random variables in the range $\left[-\sqrt{3},\sqrt{3}\right]$. In addition, consider the random variable $Z_{i}$, where $Z_{i}=1$ with probability *p* and $Z_{i}=0$ with probability 1−p. We define $MIX_{1D-RGB}\left(p\right)$ as follows:

(1) $$MIX_{1D-RGB}{\left(p\right)}_{i,k}=\left(1-Z_{i}\right)X_{i}+Z_{i}Y_{i},$$

with *k*, the different variates of the signal, 1≤k≤q; see an example in Figure 1.

#### 2.2.2. MIX Process for RGB Images

In order to evaluate our algorithms on RGB images with different controlled irregularity levels, we introduce a family of two-dimensional MIX processes for RGB images, $MIX_{2D-RGB}\left(p\right)$, based on the two-dimensional version for grayscale images [9]. We define the MIX process for RGB images as follows: consider $X_{i,j}=\sin\frac{2\pi i}{12}+\sin\frac{2\pi j}{12}$ to be a sinusoidal image and $Y_{i,j}$ an image composed of uniformly distributed white noise pixels in the range $\left[-\sqrt{3},\sqrt{3}\right]$. In addition, consider the random variable $Z_{i,j}$, where $Z_{i,j}=1$ with probability *p* and $Z_{i,j}=0$ with probability 1−p. We define $MIX_{2D-RGB}\left(p\right)$ as follows:

(2) $$MIX_{2D-RGB}{\left(p\right)}_{i,j,k}=\left(1-Z_{i,j}\right)X_{i,j}+Z_{i,j}Y_{i,j},$$

with *k* being the different channels of the image, 1≤k≤3.

**Figure 2:**
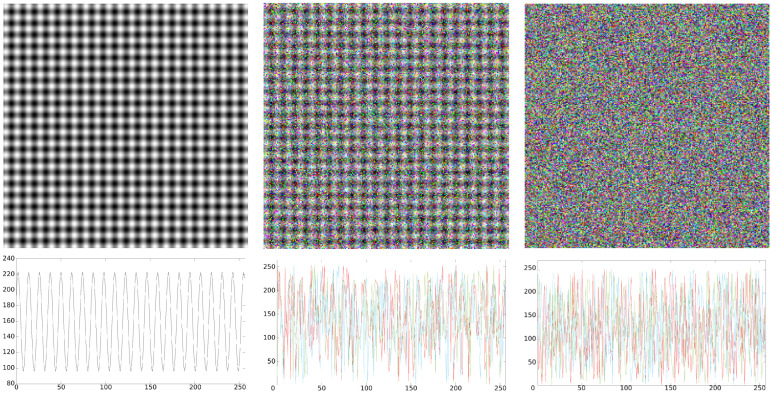
First row: 256×256 color images generated by a MIX process. *p* varies from 0 to 1 with a step of 0.5 (from left to right). Second row: pixel values of the first row from each image presented above (256 pixels). The pixels from the three color channels are presented. Note that, for *p* = 0, the channels merge into the others to form a periodic, regular sine wave. When *p* increases, the signal becomes more irregular.

By modifying the value of *p*, we can generate different levels of spatial regularity. For p=0, the generated image will present a periodic, regular pattern. For p=1, the pixel values of the resulting image will be completely random, the image will therefore be highly irregular; see Figure 2. In other words, a MIX process for RGB images is defined here as a process of three variates, with each variate being a different MIX process.

### 2.3. Pre-Processing of the Images

With respect to reducing the computation time, the images from the biomedical and generic databases have been cropped from the center to form 50×50 and 100×100 images. The values of the pixels have been converted to double and normalized. The mean and standard deviation of each image were, thus, respectively, defined as μ=0 and σ=1.

### 2.4. Entropy Methods

One of the goals of this paper is to compare texture RGB image classification results using different entropy methods, with those given by deep learning methods. Therefore, we extracted the entropy from images using the algorithms described in the following sections.

#### 2.4.1. Sample Entropy

One-dimensional approximate entropy ($ApEn_{1D}$ [2]) has shown promising results in quantifying irregularity of medical signals. Nevertheless, $ApEn_{1D}$ shows a higher similarity degree than expected, and it lacks relative consistency. Therefore, it is biased. To overcome these drawbacks, sample entropy ($SampEn_{1D}$) has been proposed in 2000 [3] and has been used in many biomedical signal processing applications. For a time series, sample entropy is the negative natural logarithm of the conditional probability that two sequences similar for *m* points remain similar at the next point, where self-matches are not included in calculating the probability. The similarity of vectors is based on the Heaviside function. A lower value of sample entropy indicates more self-similarity in the time series. $SampEn_{1D}$ is less dependent on signals’ length and more consistent than $ApEn_{1D}$. Sample entropy has been recently extended to its bi-dimensional version to process grayscale images: $SampEn_{2D}$ [9]. The drawback of both $SampEn_{1D}$ and $SampEn_{2D}$ is that they can present undefined values because of abrupt changes caused by the two-state classifier, i.e., the Heaviside function, which decides whether two vectors match each other.

#### 2.4.2. Fuzzy Entropy

Fuzzy entropy ($FuzEn_{1D}$) is a set of statistics that has been developed in 2007 [4] to avoid the problem of undefined values in $SampEn_{1D}$, by using the concept of “fuzzy sets” (introduced in 1965 [31]). In the physical world, boundaries between classes may be ambiguous, and it is difficult to determine whether an input pattern fully belongs to a class. With fuzzy entropy, we calculate a similarity degree of two vectors through a fuzzy function, which makes it possible to emancipate from the problem of hard boundaries. Therefore, for fuzzy entropy, the computation steps are similar to that of sample entropy, except that a fuzzy function (an exponential function in our work) is used for the measurement of vectors’ similarity. The bi-dimensional version of fuzzy entropy has also been proposed: $FuzEn_{2D}$ [10]. With this new measure, it was possible to extract texture information from biomedical grayscale images, such as skin texture images [10]. Very recently, entropy has been studied on RGB images and a new measure has been introduced: $FuzEn_{RGB}$ [22].

#### 2.4.3. Multiscale Entropy

The above-mentioned entropy measures have been extended to analyze data on several scale factors through their multiscale versions. Multiscale entropy measures quantify the complexity of data as their irregularity over several scale factors. The entropy is evaluated at multiple scale factors, τ, on coarse-grained signals or images constructed from the original signal or image. This creates an entropy feature vector that represents the complexity of the system. Multiscale entropy values for 1D signals, as defined by Costa et al. in 2002 [32], will decrease for completely random signals (such as white noise), and will show higher entropy values for correlated signals over several scale factors. The same feature is found for bi-dimensional multiscale entropy applied to grayscale images [8].

#### 2.4.4. Multivariate Entropy

Before the work of Ahmed et al. [5], no entropy measure existed for multivariate signals that are often encountered in biological systems. The variables were all treated separately, even for systems where the different channels were statistically dependent and correlated. Multivariate sample entropy (MSampEn) and its extension to multivariate multiscale sample entropy (MMSE) [5] analyze the entropy of each data channel separately in a rigorous and unified way. The two methods have already shown promising results in the classification of real-world multichannel data and have been extended to multivariate multiscale fuzzy entropy (MMFE) ([6,7]).

#### 2.4.5. New Introduced Methods: Multivariate Sample and Fuzzy Entropy Measures for RGB Images

The new entropy measures that we propose are the multivariate sample entropy for RGB images ($MSampEn_{RGB}$) and the multivariate fuzzy entropy for RGB images ($MFuzEn_{RGB}$), and their multiscale versions.

The calculation of the multivariate entropy for RGB images is inspired from multivariate embedding theory. The method represents a natural extension of multivariate sample entropy for 1D signals. The adaptations are mainly related to the construction of the composite delay vectors that are bi-dimensional (construction from an image and not from a signal of 1D-data). The computation of the distance between two embedding vectors is also modified, as well as the calculation of the frequency of occurrence where the bi-dimensional version of the data has to be taken into account (see below).

For a *q*-variate matrix I with *W* width and *H* height, ${\left\{x_{i,j,k}\right\}}_{i,j=0}^{W,H},k=0,1,\ldots ,q-1$, the multivariate sample entropy for RGB images is calculated according to the following steps:

1. Form $N_{m}=\left(W-n\right)\times \left(H-n\right)$ composite delay vectors $X_{m}\left(i,j\right)\in R^{m}$, where $m=\Sigma_{k=1}^{q}m_{k}$, i=0,1,…,W−n−1, j=0,1,…,H−n−1, $M=\left[m_{1},m_{2},\ldots ,m_{q}\right]\in R^{q}$ is the embedding vector, and n=max{M}. The composite delay vector $X_{m}\left(i,j\right)$ is determined as follows:
  (3) $$\begin{array}{cc} X_{m}\left(i,j\right)=[ & x_{i,j,1},x_{i+1,j,1},\ldots ,x_{i+m_{1}-1,j,1},x_{i,j+1,1},x_{i+1,j+1,1},x_{i+m_{1}-1,j+1,1},\ldots ,\\ & x_{i,j+m_{1}-1,1},x_{i+1,j+m_{1}-1,1},\ldots ,x_{i+m_{1}-1,j+m_{1}-1,1},\\ & x_{i,j,2},x_{i+1,j,2},\ldots ,x_{i+m_{2}-1,j,2},x_{i,j+1,2},x_{i+1,j+1,2},\ldots ,x_{i+m_{2}-1,j+1,2},\ldots ,\\ & x_{i,j+m_{2}-1,2},x_{i+1,j+m_{2}-1,2},\ldots ,x_{i+m_{2}-1,j+m_{2}-1,2},\ldots ,\\ & x_{i,j,q},x_{i+1,j,q},\ldots ,x_{i+m_{q}-1,j,q},x_{i,j+1,q},x_{i+1,j+1,q},\ldots ,x_{i+m_{p}-1,j+1,q},\ldots ,\\ & x_{i,j+m_{p}-1,q},x_{i+1,j+m_{q}-1,q},\ldots ,x_{i+m_{q}-1,j+m_{q}-1,q}]. \end{array}$$
2. Define the distance between any two vectors $X_{m}\left(i,j\right)$ and $X_{m}\left(a,b\right)$, where a=0,1,…,W−n−1, b=0,1,…,H−n−1 and (i,j)≠(a,b), as the Chebychev or maximum norm distance between two vectors, that is,
  (4) $$\begin{array}{c} d\left[X_{m}\left(i,j\right),X_{m}\left(a,b\right)\right]=max_{e,f\in 0,\ldots ,m-1,g\in 0,\ldots ,q-1}\left\{\right|x\left(i+e,j+f,g\right)-\\ x(a+e,b+f,g)|\}. \end{array}$$
3. For a given composite delay vector $X_{m}\left(i,j\right)$ and a threshold *r*, count the number of instances $P_{i,j}$ for which $d[X_{m}\left(i,j\right),X_{m}\left(a,b\right)]\leq r,\left(i,j\right)\neq \left(a,b\right)$; then, calculate the frequency of occurrence:
  (5) $$B_{i,j}^{m}\left(r\right)=\frac{1}{N_{m}-n-1}P_{i,j},$$
  and define
  (6) $$B^{m}\left(r\right)=\frac{1}{N_{m}-n}\Sigma_{i=1;j=1}^{i=W-n;j=H-n}B_{i,j}^{m}\left(r\right).$$
4. Extend the dimension of the multivariate delay vector in Equation (3). This can be performed in *p* different ways, as from a space with the embedding vector $M=[m_{1},m_{2},\ldots ,m_{k},\ldots ,m_{q}]$ the system can evolve to any space for which the embedding vector is $\left[m_{1},m_{2},\ldots ,m_{k}+1,\ldots m_{q}\right]$. Thus, a total of $q\times (N_{m}-n)$ vectors $X_{m+1}\left(i,j\right)\in R^{m+1}$ are obtained, where $X_{m+1}\left(i,j\right)$ denotes any embedded vector upon increasing the embedding dimension from $m_{k}$ to $m_{k}+1$ for a specific variable *k*.
5. For a given $X_{m+1}\left(i,j\right)$, calculate the number of vectors $Q_{i,j}$, such that $d[X_{m+1}\left(i,j\right),$$X_{m+1}\left(a,b\right)]\leq r,\left(i,j\right)\neq \left(a,b\right)$; then, calculate the frequency of occurrence:
  (7) $$B_{i,j}^{m+1}\left(r\right)=\frac{1}{q\times (N_{m}-n)-1}Q_{i,j},$$
  and define
  (8) $$B^{m+1}\left(r\right)=\frac{1}{q\times (N_{m}-n)}\Sigma_{i=1;j=1}^{i=p(W-n);j=p(H-n)}B_{i,j}^{m+1}\left(r\right).$$
6. Finally, for a tolerance level *r*, estimate the multivariate sample entropy as
  (9) $$MSampEnRGB\left(I,M,r\right)=-\ln\left[\frac{B^{m+1}\left(r\right)}{B^{m}\left(r\right)}\right].$$

For the multivariate fuzzy entropy, the process is similar, except for steps 3, 5, and 6. They are, respectively, replaced by the following three steps:

3. For a given composite delay vector $X_{m}\left(i,j\right)$, a threshold *r* and a fuzzy power *s*, compute the degree of similarity $D_{ij,ab}^{m}$:
  (10) $$D_{ij,ab}^{m}=\exp\left(\frac{-{\left(d\left[X_{m}\left(i,j\right),X_{m}\left(a,b\right)\right]\right)}^{s}}{r}\right).$$
  Then, the function $\Phi^{m}$ is defined as follows:
  (11) $$\Phi^{m}\left(s,r\right)=\frac{1}{N_{m}-n}\Sigma_{i=1,j=1}^{i=H-n,j=W-n}\Phi_{i,j}^{m}\left(r\right),$$
  where $\Phi_{i,j}^{m}\left(r\right)$ is the average of all the similarity degrees of a given composite delay vector $X_{m}\left(i,j\right)$.
5. For a given composite delay vector $X_{m+1}\left(i,j\right)$, a threshold *r* and a fuzzy power *s*, compute the degree of similarity $D_{ij,ab}^{m+1}$:
  (12) $$D_{ij,ab}^{m+1}=\exp\left(\frac{-{\left(d\left[X_{m+1}\left(i,j\right),X_{m+1}\left(a,b\right)\right]\right)}^{s}}{r}\right).$$
  Then, the function $\Phi^{m+1}$ is defined as follows:
  (13) $$\Phi^{m+1}\left(s,r\right)=\frac{1}{q\times (N_{m}-n)}\Sigma_{i=1,j=1}^{i=p(H-n),j=p(W-n)}\Phi_{i,j}^{m+1}\left(r\right),$$
  where $\Phi_{i,j}^{m+1}\left(r\right)$ is the average of all the similarity degrees of a given composite delay vector $X_{m+1}\left(i,j\right)$.
6. Finally, for a tolerance level *r* and a fuzzy power *s*, estimate the multivariate fuzzy entropy as
  (14) $$MFuzEnRGB\left(I,M,r,s\right)=-\ln\frac{\Phi^{m+1}\left(s,r\right)}{\Phi^{m}\left(s,r\right)}$$

### 2.5. Deep-Learning Methods

Deep learning employs neural networks that consist of several layers. The size, number, and order of these layers create different network architectures. For image related tasks such as object detection, recognition, and image segmentation, convolutional neural networks (CNN) have proven to be very effective. A typical CNN employs three distinct layer types namely convolutional, pooling, and dense layers. The number and size of these layers vary with different network architectures.

The first layers of a CNN are designed to extract high-level features within the image. The first layer is typically a convolutional layer that sweeps a kernel filter over the image producing a feature map output. The output of the convolutional layer(s) is passed to a pooling layer. Pooling layers perform an aggregation function. Through this aggregation, the dimensions of the feature vectors are reduced. Depending on the network architecture, several convolution and pooling layers may be employed. The combination of these layers is used to extract the features of the image, which are then passed to the classification layers that form the final layers of the network. These classification layers consist of flatten, dense, and softmax activation. The flatten layer converts the feature maps created by the convolutional and pooling layers into a one-dimensional vector that can be processed by the fully connected layers. The fully connected layers is composed of neurons that are connected to all the neurons in the previous layer. To create a classification output a softmax layer is used. The softmax function takes the output vector from the fully connected layer and compresses it to a vector of zero-to-one values. This allows the network to produce probability scores from 0 to 1 representing the probability that the input image contains the given class.

Several studies describing deep learning theory and architectures more thoroughly have recently been published [20,23,33,34]. From the deep learning concept, several variants have been proposed, as described thereafter.

#### 2.5.1. Full Learning (or End-to-End Learning)

In full learning, the feature extraction layers of the model are trained to learn to extract the best features from the images that will be given to the classification layers. The latter are trained to extract the probability of those features belonging to each class. This process can, therefore, be time-consuming and computationally intensive because of all the training phases. In addition, it requires a large dataset to avoid overfitting problems, which is not usually the case in biomedical databases. An advantage of end-to-end fully trained CNN is that the extracted features will be fully adapted to the database. In this work we constructed a 10-layer CNN by alternating convolution and pooling layers for the feature extraction phase and flatten and dense layers for the classification phase (see Table 2).

#### 2.5.2. Transfer Learning

Transfer learning allows us to make use of an existing pretrained network to reduce the training time and computation. When using a pretrained network, the weights of the feature extraction layers of the network are already defined as the network has been trained on a different very large dataset such as ImageNet [35] to adapt the network to the specific task. The classification head is generally removed to use the model as a feature extractor. The features can then be introduced to a machine learning algorithm (e.g., support vector machine (SVM) [36]), for which the training time is significantly less than training the entire network. Based on its good classification results in addition to reduced computation time with reference to the literature [37], we chose to use a model of residual network with 50 layers (ResNet50) [17], pre-trained on the ImageNet dataset, to extract features from the images. Those features were then given to a SVM machine learning model for classification.

#### 2.5.3. Fine Tuning

Fine tuning is a method that takes characteristics from both of the above-mentioned methods. A pre-trained model is used, but the feature extraction layers are frozen to tune the weights of the last fully connected classification layers. This type of pre-trained networks is often used in texture images classification because it does not need any training phase to extract feature vectors from the images and, therefore, a large amount of training data is not required. Pre-trained models, as used in transfer learning and fine tuning, have the advantages of being easy to implement while using a trusted and proven model. Moreover, the computational cost is relatively low, compared to end-to-end fully-trained convolutional networks. This is the reason why they have already been used in many biomedical applications (e.g., diagnosis of leukemia in blood slides [38]). For this method, we use the same ResNet50 model as the one mentioned above to extract the features of the images, but the classification is performed with fully connected layers.

## 3. Results and Discussion

### 3.1. MIX Process

Images with 100×100 pixels have been generated by a $MIX_{RGB}$ process. Multivariate fuzzy and sample entropy ($MSampEn_{RGB}$ et $MFuzEn_{RGB}$) have been computed on these images. The algorithms have been evaluated for multiple parameter combinations: M=[111] and r=0.02 or r=1, M=[222], and r=0.02 or r=1. For $MFuzEn_{RGB}$, the fuzzy power is s=2.

**Figure 3:**
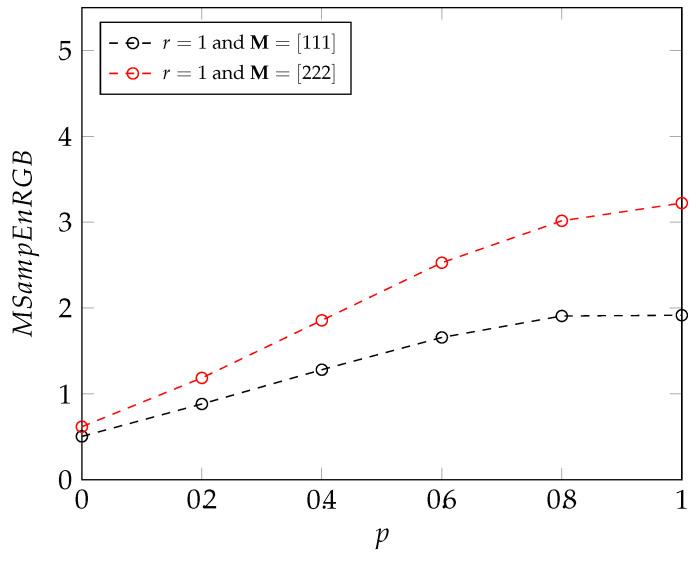

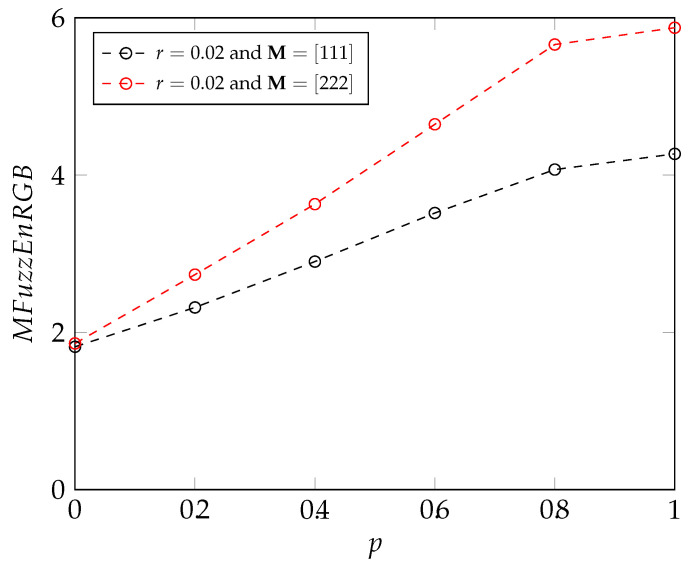
Multivariate sample and fuzzy entropy values on RGB images created with MIX processes, *p* varying from 0 to 1 with a step of 0.2.

The results obtained are as expected: an increase in the entropy value is associated with an increase in the irregularity of the image, that is induced by an increasing value of *p* (see Figure 3). With these parameters, the results show that the new entropy measures can be used to properly assess the degree of irregularity of an image. We can also deduce from these results that, for the MIX process, **M** =[222] gives us a more accurate estimation of the irregularity of an image than **M** =[111], since **M** =[222] leads to better differentiation between images of two consecutive irregularity than **M** =[111].

### 3.2. Texture Image Classification Results

Entropy associated with machine learning and deep learning algorithms have been used on the previously mentioned databases. All the entropy extractions have been performed on the Epistroma database. Images converted to grayscale have been processed with the univariate multiscale entropy measures and RGB images have been processed with the univariate multiscale entropy measures [22] and the new multivariate entropy measures. The metric used to compare the different results is the accuracy. The latter refers to the rate of good classification of the algorithm, corresponding to the following equation:

(15) $$\mathrm{accuracy}=\frac{\mathrm{Number}\;\mathrm{of}\;\mathrm{correctly}\;\mathrm{classified}\;\mathrm{images}}{\mathrm{Number}\;\mathrm{of}\;\mathrm{images}\;\mathrm{in}\;\mathrm{total}}\times 100.$$

The accuracy is then averaged over five random splits between training (75%) and test (25%). The best classification results have been selected after a phase of parameters optimization, for each database and for each method independently. The parameters are *m* and *r* for the sample entropy; *m*, *r*, and *s* for the fuzzy entropy; M and *r* for the multivariate sample entropy; and M, *r*, and *s* for the multivariate fuzzy entropy. For all the multiscale entropy calculations, a maximum scale factor τ=10 has been considered.

The results of the classification from machine learning (SVM classifier) used on the univariate entropy extractions are presented in Table 3. The results of the classification from machine learning (SVM classifier) used on the multivariate entropy extractions are presented in Table 4. From these entropy-based methods, we observe an improvement of the accuracy when the algorithms are applied to color images, compared to when they are used for grayscale images. Moreover, the results are better when the multivariate sample entropy algorithms are used instead of univariate sample entropy algorithms. We do not observe an improvement of the fuzzy entropy algorithms with the multivariate version. We also note a large improvement when we increase the size of the images.

Finally, the results of the classification from deep learning are presented in Table 5. We observe that deep learning is capable of classifying with good accuracy biomedical and generic texture RGB images, even for small-size images (50×50 pixels). Nevertheless, some architectures are not adapted to all types of databases. It is the case for the end-to-end fully trained network (10-layer CNN), that gives the worst classification results, especially when the database is composed of lots of classes (as it is the case for the Alot database that contains 250 classes). This result indicates that the training of the network on a small database will not give a representation of the image by features precise enough to differentiate a large number of classes. The best classification results are obtained on the networks trained on very large databases.

In this paper, we proposed a new entropy-based measure to process RGB images. It is based on a multivariate entropy approach. The results allow for extracting information from color image texture to serve classification purposes. Color and texture play a key role in many applications and are a hot topic in computer vision and pattern recognition [20]. Two main classes can be identified for color texture classification: the hand-crafted (traditional) methods and the data-driven (i.e., deep learning) ones. In this work, we compared classification results obtained with these two classes, having the traditional methods being based on entropy measures.

Our results show that, for the databases used and when the same type of classifier—SVM on both feature extractions phases (entropy and deep learning features)—is chosen, deep learning outperforms entropy for the classification tasks. These results are in accordance with other studies, performed on other databases and with other hand-crafted methods [21]. This means that the deep learning extraction gives a more precise representation of the image, with relatively little intervention from the end user. However, this representation is not totally linked to the texture of the image and takes into consideration a large number of features, unlike the entropy measures that are specific to texture analysis. Moreover, the data-driven method relies on layers that contain free parameters. The latter have to be set by a compulsory step, the training step. This is why, in the last few years, CNN—which has received increasing attention—has often been used with pre-trained networks. In contrary, the hand-crafted methods do not require any training. Moreover, they are well-established and transparent. It has been shown by others that when direction and temperature of the light change simultaneously, hand-crafted descriptors can perform better than learned descriptors [21]. Finally, even if the deep-learning methods can lead to very good performances in classification studies, they certainly are not the panacea for all problems [23]; among others, they still have the drawback of being black boxes to the end user. This can be an issue for some application domains, particularly when medical data are involved. Moreover, for some tasks (e.g., classification of images from very small databases, often encountered in the medical field), the use of deep learning is excessive and the size of the database will be insufficient to train the model. The future would perhaps be to aggregate descriptors resulting from the combination of hand-crafted methods and deep learning ones, as recently proposed [39,40].

## 4. Conclusions

The image processing domain has been subject to drastic changes with the recent development of data-driven intelligence. We have seen that some traditional computer vision methods are being replaced by deep learning approaches because the aforementioned are often more efficient. However, we can still find many applications where there is still advantages from years of work of hand-crafted methods. Further improvements of these traditional methods are essential to address modern computer vision issues. Our multivariate entropy algorithms show promising results in the analysis of the texture for RGB biomedical images. Other studies could be conducted to extend these methods to other color spaces. The future step would perhaps be aggregating descriptors that result from the combination of hand-crafted methods and deep learning ones.

## Figures and Tables

**Table 1:** Databases used in this study. All databases contain RGB images.

Dataset	Subject	Classes	Images per Classes (Mean)	Total Images	Images Size (px)	Year	Reference
Epistroma	Histological images of colorectal cancer	2	688	1376	172 × 172 to 2372 × 2372	from 1989 to 1998	[25]
KTH-TIPS2-a	Mixed	11	432	4608	200 × 200	2006	[27]
ALOT	Mixed	250	100	25,000	1536 × 1024	2009	[29]

**Table 2:** Architecture of the proposed CNN.

10-Layers CNN			
**Layer**	**Input**	**Output**	**Parameters**
Rescaling	image size	(224, 224, 3)	0
Conv2D	(224, 224, 3)	(222, 222, 32)	896
MaxPooling2D	(222, 222, 32)	(111, 111, 32)	0
Conv2D	(111, 111, 32)	(109, 109, 64)	18,496
MaxPooling2D	(109, 109, 64)	(54, 54, 64)	0
Conv2D	(54, 54, 64)	(52, 52, 64)	36,928
MaxPooling2D	(52, 52, 64)	(26, 26, 64)	0
Flatten	(26, 26, 64)	(1, 43,264)	0
Dense	(1, 43,264)	(1, 128)	5,537,920
Dense	(1, 128)	(1, number of classes)	258

**Table 3:** Average accuracy of machine-learning classification (SVM) on univariate entropy extractions results of 50×50 pixels images from the Epistroma database. For sample entropy, the parameters are: m=1, r=0.02. For fuzzy entropy, the parameters are m=4, r=0.24, and s=9. These parameters are those that give the best results on the database used.

Univariate sample entropy (for grayscale images):	67.02%
Univariate multiscale sample entropy (for grayscale images):	69.06%
Univariate fuzzy entropy (for grayscale images):	62.85%
Univariate multiscale fuzzy entropy (for grayscale images):	66.95%
Univariate fuzzy entropy (for RGB images):	67.75%
Univariate multiscale fuzzy entropy (for RGB images):	69.10%

**Table 4:** Average accuracy of SVM classifier performed on multivariate multiscale entropy extractions of RGB images from the Epistroma dataset. The sizes 50×50 pixels and 100×100 pixels have been considered. For sample entropy, the parameters are M=[222], r=0.15. For fuzzy entropy, the parameters are M=[222], r=0.15 and s=2. These parameters are those that give the best results on the database used.

Size of the images	50 × 50	100 × 100
Multivariate multiscale sample entropy RGB	70.64%	78.11%
Multivariate multiscale fuzzy entropy RGB	66.45%	72.96%

**Table 5:** Deep learning classification results for 50×50 pixels RGB images. The best results are shown in bold.

	Epistroma	KTH-TIPS2-a	Alot
10-layers CNN	91.03%	85.32%	67.25%
Resnet50 with fully connected layer	**93.05%**	94.98%	81.04%
Resnet50 with SVM classification	92.22%	**96.54%**	**81.08%**

## Data Availability

Not applicable.
